# Preoperative bevacizumab combined with letrozole and chemotherapy in locally advanced ER- and/or PgR-positive breast cancer: clinical and biological activity

**DOI:** 10.1038/sj.bjc.6604741

**Published:** 2008-10-21

**Authors:** R Torrisi, V Bagnardi, A Cardillo, F Bertolini, E Scarano, L Orlando, P Mancuso, A Luini, A Calleri, G Viale, A Goldhirsch, M Colleoni

**Affiliations:** 1 Department of Medicine, Research Unit of Medical Senology, European Institute of Oncology Milan, via Ripamonti 435, 20141 Milan, Italy; 2 Department of Medicine, European Institute of Oncology Milan, via Ripamonti 435, 20141 Milan, Italy; 3 Division of Epidemiology and Biostatistics, European Institute of Oncology Milan, via Ripamonti 435, 20141 Milan, Italy; 4 Department of Statistics, University of Milan Bicocca, Piazza dell’Ateneo Nuovo, 1, 20126, Milan, Italy; 5 Frontier Science and Technology Research Foundation, Southern Europe, European Institute of Oncology Milan, Milan, Italy; 6 Division of Hematology Oncology, European Institute of Oncology Milan, via Ripamonti 435, 20141 Milan, Italy; 7 Division of Senology, European Institute of Oncology Milan, via Ripamonti 435, 20141 Milan, Italy; 8 Division of Pathology, European Institute of Oncology Milan, via Ripamonti 435, 20141 Milan, Italy; 9 University of Milan, School of Medicine via Festa del Perdono 7, 20135 Milan, Italy

**Keywords:** bevacizumab, chemoendocrine therapy, preoperative therapy, endocrine-responsive breast cancer

## Abstract

The antiangiogenic agent bevacizumab showed synergistic effects when combined with chemotherapy in advanced breast cancer. We presently investigated the activity of bevacizumab in combination with chemotherapy, including capecitabine and vinorelbine, and endocrine therapy, including letrozole (+triptorelin in premenopausal women), as primary therapy for patients with ER and/or PgR ⩾10% T2–T4a-c, N0–N2, M0 breast cancer. Biological end point included the proliferative activity (Ki67), whereas clinical end points were clinical response rate, pathological complete response (pCR) and tolerability. Circulating endothelial cells (CECs) and their progenitors, as surrogate markers of antiangiogenic activity, were measured at baseline and at surgery.Thirty-six women are evaluable. A clinical response rate of 86% (95% CI, 70–95) and no pCR were observed; Ki67 was significantly decreased by 71% (interquartile range, −82%, −62%). Toxicity was manageable: two grade 3 hypertension, four grade 3 deep venous thrombosis and no grade >2 proteinuria were observed. Treatment significantly decreased the percentage of viable CECs and prevented the chemotherapy-induced mobilisation of circulating progenitors. Basal circulating progenitors were positively associated with clinical response. In conclusion, bevacizumab is feasible and active in association with primary chemoendocrine therapy for ER-positive tumours in terms of proliferation inhibition, clinical response and antiangiogenic activity.

Preoperative treatment of endocrine-responsive breast cancer is a matter of debate within the scientific community. The striking improvement in terms of pathological complete response (pCR) obtained in endocrine-unresponsive tumours with new schedules of anthracyclines and taxanes has had a lesser impact in ER- and PgR-positive tumours, which obtained pCR up to 10% (Mazouni *et al*, 2007).

On the other hand, endocrine therapy, although associated with a high clinical response rate, almost rarely yields pCR (up to 3–6%) (Kaufmann *et al*, 2006). The association of chemoendocrine therapy, not systematically investigated in an appropriate population, has obtained discouraging results too (von Minckwitz *et al*, 2001; Bottini *et al*, 2005).

We have investigated earlier the activity of the combination of a non-anthracycline- and taxane-based chemotherapy with endocrine therapy in a series of patients with ER- and PgR-positive tumours (Torrisi *et al*, 2008). Chemotherapy included six courses of capecitabine and oral vinorelbine administered in association with letrozole (+3-monthly triptorelin, if premenopausal). The results were rather disappointing, with a response rate of 62% and no patient obtaining a pCR (Torrisi *et al*, 2008).

Angiogenesis represents a key process in the development and growth of cancer cells at any stage (Folkman, 1971). Vascular endothelial growth factor (VEGF) is the most potent and specific angiogenic factor and has been identified as a crucial regulator of both normal and pathological angiogenesis (Banerjee *et al*, 2007). Vascular endothelial growth factor acts as a mitogen for vascular endothelial cells and stimulates the secretion of enzymes involved in extracellular matrix degradation. Moreover, VEGF stimulates paracrine and autocrine signalling in endothelial cells (Banerjee *et al*, 2007). The recombinant humanised anti-human VEGF monoclonal antibody (rhuMAb, bevacizumab) inhibits several activities of VEGF, including endothelial cell growth, vascular permeability and angiogenesis (Kim *et al*, 1993; Willett *et al*, 2004). Bevacizumab has shown substantial activity in breast cancer and synergism with some chemotherapeutic agents (Sweeney *et al*, 2001; Cobleigh *et al*, 2003). The combination of bevacizumab and vinorelbine was investigated in a phase II study in patients with refractory advanced breast cancer, and a 31% objective response rate was observed (Burstein *et al*, 2002). In a phase III randomised study in breast cancer patients pretreated with anthracyclines and taxanes, the addition of bevacizumab significantly increased the response rate as compared with capecitabine alone (Miller *et al*, 2005). In untreated metastatic patients, the association of bevacizumab and paclitaxel significantly improved either response rate or progression-free survival as compared with paclitaxel alone (Miller *et al*, 2007). On the other hand, oestrogens are potent modulators of angiogenesis. It is thus conceivable that the combination of an antiangiogenic agent and a hormonal manipulation may result in an increased antitumour activity on an endocrine-sensitive tumour. In preclinical models, oestrogens increase endothelial cell proliferation and migration, whereas the effect of aromatase inhibition on angiogenic factors is less clear. In fact, in preclinical models, aromatase inhibitors downregulate VEGF expression, whereas no significant change of serum VEGF has been observed after preoperative anastrozole (Traina *et al*, 2007; Banerjee *et al*, 2008). Preclinical data also suggest that VEGF is involved in precocious stages of angiogenesis (Relf *et al*, 1997). It may thus be speculated that the earlier the angiogenic pathway is blocked, the greater the clinical effect that may be expected. Given the encouraging results obtained by the combination of capecitabine and vinorelbine in advanced breast cancer and the synergism with bevacizumab shown by both drugs, we thereby decided to investigate the combination of bevacizumab, capecitabine and vinorelbine and endocrine therapy in patients with ER- and/or PgR-positive locally advanced breast cancer. We defined as the principal measure of activity, the decrease of proliferation by Ki67, which has been shown to correlate better with prognosis than the absolute baseline value (Burcombe *et al*, 2005; Dowsett *et al*, 2005). Clinical activity in terms of either objective response rate, pCR or the tolerability of the combination was also evaluated.

Finally, with the aim of identifying putative predictors and biomarkers of antiangiogenic activity, we determined baseline and post-treatment levels of circulating endothelial cells (CECs), either viable or apoptotic, and their progenitors and investigated their correlation with clinical outcome variables.

## Materials and methods

### Patients

Patients with histologically proven T2–T4a-c, N0–N2, M0, ER and/or PgR ⩾10% and HER2-negative breast cancer were considered eligible for the study. Eligibility criteria also included no earlier chemotherapy/hormonotherapy, Eastern Cooperative Oncology Group performance status 0–2, measurable lesions, age between 18 and 70 years, white blood cells ⩾3000 per mm^3^, platelets ⩾100 000 per mm^3^, aspartate aminotransferase, alanine aminotransferase, ⩽2.5 × upper limit of normal and bilirubin ⩽3 mg per 100 ml.

Patients with cardiac disease (congestive heart failure, history of myocardial infarction within the previous 3 months), severe vascular disease or uncontrolled concomitant infections were excluded. In addition, patients with a prior history of bleeding diathesis or coagulopathy, including deep venous thrombosis (DVT) or pulmonary embolism, recent (within last 6 months) or current history of gastrointestinal bleeding and current use of full-dose or parenteral anticoagulants or chronic daily treatment with aspirin (greater than 325 mg day^−1^) were excluded as were patients with 24 h urine protein greater than or equal to 500 mg or any active primary renal disease (excluding infection).

Patients had baseline liver and renal function tests, electrolyte studies and complete blood count and urine analysis performed within 2 weeks of inclusion in the study. In addition, bilateral mammography and breast ultrasound, chest X-ray, abdominal ultrasound, bone scan or FDG-PET, serum CA 15.3 determination and electrocardiography were performed within 2 weeks from the start of treatment. Before starting the treatment and providing a signed informed consent, patients were submitted to a trucut to obtain a tumour sample to be stored for gene profiling and further molecular determination and a blood sample for the determination of circulating biomarkers (VEGF, CEC and their progenitors). Blood samples were also obtained before (day −1) and immediately after surgery (week +1).

Willing patients were submitted to a trucut after 3 weeks (first cycle) for assessment of early change in Ki67.

Written informed consent from all patients was obtained. The protocol was approved by the Ethical Committee.

### Treatment

Patients received chemotherapy containing capecitabine 2000 mg m^−2^ orally on days 1–14, vinorelbine 20 mg m^−2^ i.v. on days 1 and 3 (Nolè *et al*, 2006), and bevacizumab 15 mg kg^−1^ i.v. on day 1 every 3 weeks for eight courses. Bevacizumab (Avastin®; Roche, Basel, Switzerland) was administered as a 90′ i.v. infusion at the first administration and as 60′ and then 30′ infusions in the following courses. Bevacizumab was provided at no cost by Roche. A central venous catheter (CVC) in the subclavian or in the jugular vein contralateral to the site of the tumour was implanted in all patients before starting bevacizumab.

Endocrine therapy consisted of letrozole 2.5 mg day^−1^. Treatment started on day 1 of the first course of chemotherapy in postmenopausal patients. In premenopausal patients, GnRH analogue (triptorelin 11.25 mg every 3 months) started on day 1 of chemotherapy and letrozole was added when oestradiol levels were in the postmenopausal range according to the IEO laboratory reference values. The median time to start of letrozole in premenopausal women was 50 days (interquartile (IQ) range, 42–69 days).

Endocrine treatment was continued until the day of surgery. Surgery was planned 4 weeks after the last dose of bevacizumab.

### Toxicity and dose modifications

Toxicity was evaluated according to NCIC-CTG 3.0 criteria by clinical and laboratory evaluations at day 21 of each cycle.

Chemotherapy was postponed by 1 week if the blood count on day 21 showed a neutrophil count <1000 per mm^3^ and/or a platelet count <100 000 per mm^3^. If on day 28 the neutrophil count was >1000 per mm^3^ and platelet count >100 000 per mm^3^, the treatment was re-administered. If after 2 weeks of treatment delay (on day 35), haematologic recovery (neutrophils >1000 per mm^3^ and platelets >100 000 per mm^3^) was not obtained, chemotherapy was discontinued.

Bevacizumab was held or definitively suspended in case of three to four haemorrhagic events, venous or arterial thromboembolic events, uncontrolled hypertension, grade 4 proteinuria, gastrointestinal perforations, wound healing complications and infusion-related allergic reactions.

### Response criteria

Tumour was evaluated at baseline by physical measurement, with callipers, of the two largest diameters and by means of mammography and ultrasound. After four and eight cycles, patients also had mammography and ultrasound breast examination to assess response. Clinical responses were evaluated according to both radiological (breast ultrasound or mammography) and clinical evaluation, by measuring the largest diameters of the tumour, and graded according to standard RECIST criteria (Therasse *et al*, 2000).

Patients with stable disease, partial remission or complete remission were candidates for four more courses of therapy. Pathological complete remissions were evaluated according to Kuerer *et al* (1999). A pCR was defined as the total disappearance of invasive tumour either in the breast or in the axilla. The presence of intraductal carcinoma qualified for pCR.

### Pathology and immunohistochemistry

All included patients had pathological evaluation performed at the EIO. Surgical specimens were extensively sampled for the evaluation of residual tumour after primary chemotherapy as published earlier.

The immunostained slides were evaluated independently by two of the authors, as reported earlier. Only nuclear reactivity was taken into account for ER, PgR and Ki67 antigens. The results were recorded as the percentage of immunoreactive cells over at least 2000 neoplastic cells.

HER2 status was defined at immunohistochemistry (IHC) as negative (faint and partial staining in >10% of cells=1+) and equivocal (faint and complete staining in >10% of cells=2+). In the latter cases, fluorescence *in situ* hybridisation (FISH) was performed to assess the amplification of the HER2 gene.

### Biological measurements

Blood samples were collected at baseline, immediately before surgery, and 1 week after surgery to estimate any change in CECs and their progenitors (CEPs).

Peripheral blood samples were collected for measurement of circulating cells by six flow cytometry. Cell suspensions were evaluated by FACSCanto (Becton Dickinson, San Jose, CA, USA). The antibodies used were CD31 and CD146 (EC marker), CD45 (pan-haematopoietic marker), CD133 (AC133, progenitor/stem cell marker), CD34 (progenitor/stem cells, EC), VEGFR-1, VEGFR-2 and VEGFR-3.

Fluorescently labelled isotype-matched IgG1 antibodies were used as control for analysis.

Appropriate analysis gates were used to enumerate viable and apoptotic CECs and CEPs.

After acquisition of at least 1 × 10^6^ cells per blood sample, analyses were considered as informative when adequate numbers of events (i.e. >100) were collected in the CEC enumeration gates. Circulating endothelial cells were defined as negative for the haematopoietic marker CD45, positive for the endothelial markers CD31 and CD146, and negative for the progenitor marker CD133. Circulating endothelial progenitor cells were depicted by the expression of CD133. 7AAD was used to gain insight into CEC/CEP viability according to Philpott *et al* (1996).

Sorted CECs, investigated by electron microscopy, were found to be bona fide endothelial cells by the presence of Weibel–Palade bodies. More than 75% of the circulating mRNAs of the endothelial-specific gene, VE-cadherin, found in the blood, were present in the sorted population. Coefficients of variation related to the CEC and CEP enumeration procedure by flow cytometry were 4±4 (intrareader), 17±4 (inter-reader) and 17±7% (variability over 0–72 h), respectively (P Mancuso *et al*, 2008, in press).

### Statistical methods

The primary objective of the study was to estimate the effect of bevacizumab on the change in tumour cell proliferation by assessment of Ki67, a biomarker of cell proliferation, in breast cancer tissue at baseline (Ki67_T0_) and after 24–28 weeks (surgical resection specimen) of treatment (Ki67_T1_). The decrease was expressed in terms of relative reduction within each patient and calculated as ((Ki67_T1_–Ki67_T0_)/Ki67_T0_) × 100.

The secondary efficacy objectives of the study were (a) to assess the effect of 24 weeks of bevacizumab on the change in tumour size, as assessed by ultrasound and mammography, and on the percentage of pCR as defined earlier; (b) to estimate any change in breast cell proliferation as measured by Ki67 in biopsies after 3 weeks; and (c) to estimate any change in CECs, in their sub-population and in the circulating progenitors, in blood at baseline, immediately before surgery, and 1 week after surgery.

According to the historical data collected at our institute on about 400 T2–T4, N0–N2, M0 breast cancer patients receiving primary chemotherapy, about 25% of patients had a relative decrease greater than 80% (Colleoni *et al*, 2004).

At the design phase, a sample size of 30 patients was planned, yielding an 80% power to detect a 50% proportion of patients with a relative decrease of Ki67 levels greater than 80%, twice as great as the proportion observed in historical controls (25%).

The Fisher's exact test and the Wilcoxon signed-rank test were used to evaluate differences in the distribution of categorical and continuous variables, respectively. The Wilcoxon signed-rank test was used to evaluate differences within the same patients of continuous variables measured at two different time points.

Subgroup analyses and/or analyses of secondary end points were exploratory in nature.

All *P*-values were two sided. The statistical analyses were run using SAS version 8.2 (SAS Institute Inc., Cary, NC, USA).

## Results

From May 2006 to January 2007, 37 patients were enrolled in the study. One patient was diagnosed with suspicious ischaemic cerebral alterations at the basal MRI and neither received bevacizumab nor was considered evaluable. Thirty-six patients were evaluated for clinical and biological end points.

Baseline characteristics of patients and tumours are reported in Table 1. Median age was 44 years (range, 30–68). Twenty-five patients (69%) were premenopausal and 11 (31%) were postmenopausal as assessed by circulating gonadotrophins and oestradiol. Eighty-six per cent of tumours were T2–T3 and 69% were clinically nodal positive (at ultrasound and/or FDG-PET).

As for protocol, all patients had ER ⩾10% tumours, whereas PgR was positive in 30 out of 36 tumours. HER2 was negative at IHC in all but one tumour, whereas FISH was negative. At baseline, Ki67 was >20% in 22 tumours (61%). At surgery, all tumours but one remained ER positive, whereas PgR switched to a negative phenotype in 25 out of 30 patients. The patient who switched to a negative ER phenotype also turned out to be HER2 positive at IHC and at FISH while remaining IHC 2+ and FISH negative at baseline.

Responses after treatment are summarised in Table 2. A complete response was observed in one patient (3%) and a partial response was observed in 30 patients (83%) with a cumulative objective response rate of 86% (95% CI, 70–95). Fourteen patients had pathological negative nodes (39%; 95% CI, 23–56). Breast-conservative surgery was feasible in 64% of patients (95% CI, 46–79) and was raised to 74%, when excluding T4 tumours, who were candidated to mastectomy irrespective of response.

In premenopausal patients, letrozole was started at a median of 50 days (IQ range, 43–69) after the first cycle of chemotherapy and bevacizumab at the achievement of oestradiol levels within the postmenopausal range. The overall median duration of letrozole was 139 days (IQ range, 119–168). The median duration of letrozole was 134 days (IQ range, 111–140) in premenopausal patients and 183 days (IQ range, 168–203) in postmenopausal patients, respectively (*P*<0.0001).

**Figure 1:**
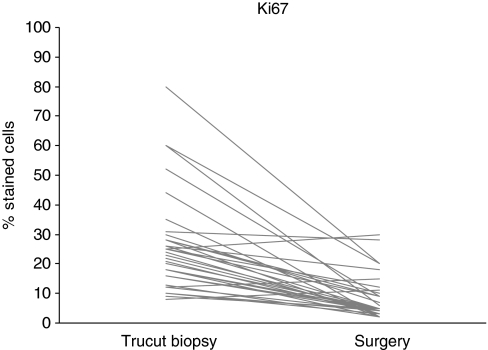
Change between pre- and post-treatment levels of Ki67 in each patient.

Median Ki67 at baseline was 23.5 (IQ range, 14.5–29), whereas after treatment, the median value was 5 (IQ range, 3–9.5%). The median percent decrease was 71% (IQ range, −82%, −62%), with *P*-value <0.0001 (Wilcoxon signed-rank test) (Figure 1). Thirty-one percent of patients (95% CI, 16–48) had a decrease ⩾80%. The only factor significantly associated with Ki67 decrease >80% was the duration of letrozole treatment, in that 50% of patients receiving letrozole for more than the median time (139 days) experienced a decrease of Ki67 >80% as compared with 12% of patients treated for a shorter time (*P*=0.03). When the analysis was performed according to the menopausal status, this association was maintained with a borderline significance in premenopausal patients (140 *vs* 122 days, *P*=0.056), whereas it was completely lost in postmenopausal patients (191 *vs* 181 days, *P*=0.45).

On the other hand, the duration of letrozole was not associated with the likelihood of obtaining a clinical response.

Ki67 was determined after 3 weeks (first cycle of bevacizumab and chemotherapy) in 17 patients. In these patients, an early significant decrease was observed (median percent decrease 65% (IQ range, 44–69%) *P*<0.001).

Toxicities grade >2 are summarised in Table 3. Bevacizumab was administered for eight courses in 26 patients. Bevacizumab was discontinued before the fourth cycle in two patients for the occurrence of DVT of subclavian (one case) and internal giugular vein (one case), in both cases corresponding to the site of insertion of the catheter of the CVC. In one further patient, bevacizumab was discontinued after the sixth cycle because of the occurrence of a seizure associated with MRI alteration referable to a leukoencephalopathy. A DVT of the giugular vein also occurred in this patient. A DVT of the femoral vein occurred in another patient after completion of the treatment before surgery. In one patient, bevacizumab was held for three cycles because of grade 3 hypertension, and two patients received six and seven cycles because of infection of the CVC and a delay in the implant of CVA, respectively. In the remaining five patients, treatment was discontinued before the eighth cycle because of grade >2 chemotherapy-related toxicity. Grade 3 hypertension was observed in two patients and grade 2 in seven patients, probably related to bevacizumab administration. One case of grade 2 proteinuria was observed that did not require drug discontinuation. Grade 1 bleeding, mainly epistaxis, was observed in five patients. No major surgical complication occurred after surgery except for one case of infection and one case of wound healing delay.

**Figure 2:**
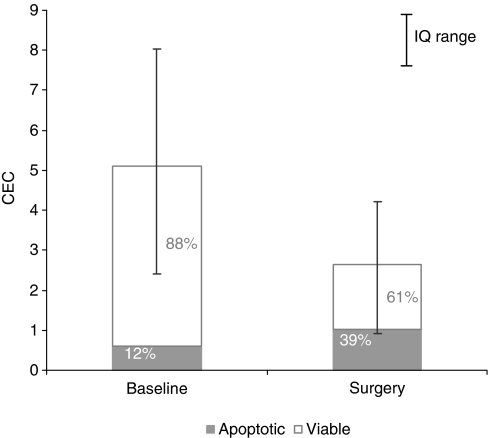
Circulating endothelial cells (CECs) at baseline and after surgery. The fraction of viable (white box) and apoptotic CECs (grey box) contributing to the absolute CEC count is shown.

Measurements of CECs and CEPs were feasible in all patients at baseline and in 29 patients at surgery, whereas only a small proportion of patients had blood samples drawn at 1 week after surgery, and statistical analyses were not performed for the latter group. Treatment did not significantly affect the total number of CEPs and CECs, although a trend towards reduction of CECs was observed (*P*=0.07). However, when different sub-populations of CECs were analysed separately, a significant increase in apoptotic CECs and a significant decrease in viable CECs was evident (Figure 2). At the same time, a significant increase in CD31+VEGFR-1+ was observed.

**Figure 3:**
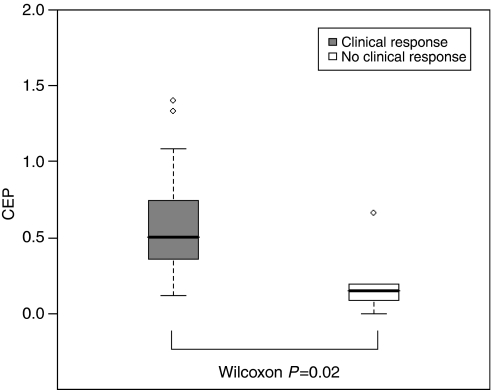
Circulating endothelial progenitor cells (CEPs) at baseline according to clinical response. Whiskers (standard span) were extended to 1.5 times the interquartile range outside the first and third quartiles. Outliers beyond the standard span were indicated with empty dots.

When we analysed the correlation of biological parameters with clinical response, we observed that higher levels of baseline CEPs were positively correlated with the likelihood of obtaining a clinical response (*P*=0.026) (Figure 3) and showed a trend towards the prediction of a Ki67 decrease >80% (*P*=0.08). No other factor significantly correlated with clinical response, the likelihood of having pathological negative nodes at surgery or of obtaining a reduction of proliferative activity >80%.

At the same time, the change of molecular parameters induced by treatment was not predictive of any clinical outcome variable except for a significant correlation between the CD31+/VEGFR-2+ sub-population of CECs and clinical response (*P*=0.017).

## Discussion

Preoperative treatment of endocrine-responsive tumours represents a challenge for medical oncologists. Although ER- and/or PgR-positive tumours respond poorly to primary chemotherapy in terms of pCR, long-term prognosis of these tumours is consistently better compared with that of ER-negative tumours (Guarneri *et al*, 2006; Colleoni *et al*, 2008). Different treatment approaches and surrogate biomarkers of activity are required in this subset of tumours.

Angiogenesis represents a key process at multiple steps in breast carcinogenesis (Folkman, 1971). Different from other molecular-targeted drugs recently recognised in the therapeutic armamentarium of the medical oncologists, antiangiogenic drugs do not work through interaction with a specific target but by interfere with a pathway that is shared by all neoplastic cells. It is thus conceivable that endocrine-responsive tumours may benefit from a disruption of the angiogenic switch. Literature data confirm that VEGF expression is a significant prognostic factor of clinical outcome, irrespective of hormone receptor status (Banerjee *et al*, 2007). In patients receiving endocrine therapy for advanced breast cancer, tissutal VEGF levels were inversely related to clinical response (Foekens *et al*, 2001; Manders *et al*, 2003).

Bevacizumab was shown to improve clinical activity of chemotherapy in advanced breast cancer, although no effect on overall survival has been observed (Miller *et al*, 2005, 2007), and the drug is currently investigated as adjuvant treatment of early breast cancer in association (concurrently or sequentially) with chemotherapy. Preclinical data suggest that the role of VEGF is crucial in the early stages of breast carcinogenesis, and the implication of an anti-VEGF therapy may be greater the earlier the treatment is introduced (Relf *et al*, 1997). The clinical and molecular activities of a single course of bevacizumab before adding chemotherapy with docetaxel were assessed in inflammatory breast cancer (Wedam *et al*, 2006). Significant decreases of VEGFR-2 and of vascular permeability (assessed by MRI parameters), correlated with clinical response, were observed (Wedam *et al*, 2006). Preliminary data on the combination of bevacizumab and chemotherapy have consistently reported a high response rate, although differences in pCR rate have been observed across studies (Lyons *et al*, 2006; Wedam *et al*, 2006; Greil *et al*, 2007).

Short-term change of proliferative activity better than baseline levels after primary chemotherapy and endocrine therapy has been associated with clinical outcome (Chang *et al*, 1999; Dowsett *et al*, 2005). In a randomised study of preoperative therapy, the 2-week decline of Ki67 was greater after anastrozole as compared with tamoxifen and the combination of both agents, mirroring the results in terms of DFS obtained in the phase III randomised trial of adjuvant therapy with the three (Dowsett *et al*, 2005). The proliferative activity at surgery was also shown as a significant independent predictor of long-term outcome after primary chemotherapy (Jones *et al*, 2007). In our study, a dramatic decrease of Ki67 was observed after treatment, with a median post-treatment value of 5%. In addition, about one-third of patients achieved a decrease >80%. Interestingly, the likelihood of achieving a greater decrease was positively correlated with the duration of endocrine treatment, underscoring the relevance of an endocrine manipulation in patients with endocrine-responsive tumours. However, given the multiplicity of treatments and the limited number of patients considered, it is not possible to assess how the single treatment contributed to this dramatic decrease of proliferative activity. Limited data are available with the combination of bevacizumab and endocrine therapy, although preliminary data show a promising activity of the combination of bevacizumab and letrozole as primary therapy in postmenopausal women with ER-positive breast cancer (Forero-Torres *et al*, 2007).

Although it has been proposed that assessing an antiangiogenic treatment by measuring tumour shrinkage may not fully reflect the antitumour activity of the drug, in this study, we observed that 86% of imaging confirmed clinical responses, a significant figure in this population of patients. In addition, the proportion of patients with pathological negative nodes is comparable with that reported in a population of ER-positive patients treated with anthracylines and taxanes containing chemotherapy (Guarneri *et al*, 2006). After treatment, 74% of T2–T3 tumours were treated with conservative surgery, which represents an appreciable figure. In an earlier study with capecitabine and oral vinorelbine in association with endocrine therapy in a similar population of patients, we observed a clinical response rate and a breast-conserving surgery rate of 62% (Torrisi *et al*, 2008). Although recognising the limitation of an indirect comparison and that other factors such as the longer duration of treatment (eight *vs* six courses) and the different routes of vinorelbine administration (intravenous *vs* oral) may have concurred with this difference, the addition of bevacizumab appeared to increase the clinical response and the breast-conserving rate as compared with chemoendocrine therapy, although not affecting the likelihood of obtaining pathological complete remission. The value of pCR as a surrogate end point of clinical outcome in ER-positive tumours after primary chemotherapy has been questioned, given the inconsistent results deriving from the analysis of retrospective series (Ring *et al*, 2004; Guarneri *et al*, 2006). The limited number of pCRs observed in both series among ER-positive tumours may account for this inconsistency, and the search for other markers to assess that the value of preoperative therapy in this sub-population is strongly encouraged.

The identification of surrogate markers of antiangiogenic activity different from standard tumour measurement appears crucial for monitoring drug efficacy and for designing an optimal integration with conventional antitumoral strategies (Bertolini *et al*, 2006). An increase of apoptotic CECs was shown to positively correlate with clinical response and clinical benefit in patients treated with metronomic chemotherapy (Mancuso *et al*, 2006). In patients treated with neoadjuvant chemotherapy, significantly higher levels of CECs were observed in patients with ER-negative as compared with ER-positive tumours; in addition, an increase in CEPs as well as a reduction of CECs was observed after neoadjuvant chemotherapy (Furstenberger *et al*, 2006). An increase in CECs also predicted a worse outcome in patients with metastatic breast cancer receiving the combination of bevacizumab and letrozole (Traina *et al*, 2007). In our study, baseline levels of CECs in patients with newly diagnosed and localised breast cancer were lower than those observed in patients with advanced disease, supporting the hypothesis that the synthesis of proangiogenic factors increases along with tumour progression (Relf *et al*, 1997).

As expected, treatment increased the proportion of apoptotic CECs while reducing the viable CECs. However, this effect was not correlated with any of the clinical outcome variables considered. A body of evidence suggests that CEPs are mobilised by chemotherapy (Bertolini *et al*, 2003; Furstenberger *et al*, 2006). As we did not observe any modulation of CEPs, we may speculate that bevacizumab prevented the chemotherapy-induced mobilisation of CEPs, as already shown in preclinical models (Shaked *et al*, 2006). On the other hand, given the positive correlation between higher baseline levels of circulating progenitors and the likelihood of obtaining a clinical response and the involvement of CEPs in neovascularisation, we may further speculate that these are patients who may benefit more from an antiangiogenic manipulation. In addition, we observed a significant correlation between the modulation of CD31+/VEGFR-2+ and the likelihood of obtaining a clinical response, in that patients who responded to treatment did not experience an increase of this sub-population of CEC. CD31+/VEGFR-2+ cells are crucial in the neovascularisation process and have recently been shown to be elevated in breast cancer patients to significantly correlate with tumour size and to rapidly decline after removal of the tumour, suggesting a possible induction by tumour-driven angiogenic stimuli (Richter-Ehrenstein *et al*, 2007).

CD31+VEGFR-1+ CECs, a sub-population that still lacks an in-depth biological characterisation, were found to be significantly increased by treatment. Further studies have been planned to better understand this phenomenon.

The manner in which bone marrow-derived progenitor cells contribute to neovascularisation either after differentiation in mature endothelial cells or after homing to sites of angiogenesis is still a matter of debate (Shaked *et al*, 2005). Our findings are consistent in showing that antiangiogenic treatment is also able to induce *in vivo* a biological response in terms of inhibition of the CEC and their progenitors involved in the angiogenic process. In addition, there is a suggestion towards the usefulness of some of these populations in monitoring biological activity, given the observed correlation with clinical response. These hypotheses should further be exploited in larger subsets of patients.

We showed that bevacizumab may also be safely administered in the neoadjuvant setting with two minor postsurgical complications, which may be attributed to the antiangiogenic treatment. The rate of bevacizumab-related adverse events was consistent with previous data, except for 11% of DVT, which was greater than that reported in the phase III trials (Miller *et al*, 2007). However, the presence of a CVC and the concurrent use of cytotoxic agents, as vinorelbine, potentially inducing an endothelial damage may have concurred with this figure. Hypertension was in most cases grade 2 and was easily managed with therapy. It required drug discontinuation in only one case, whereas proteinuria was negligible.

In conclusion, the combination of bevacizumab, endocrine therapy and tailored chemotherapy induced a high clinical response rate in ER-positive breast cancer, whereas proliferative activity of the tumour was dramatically reduced by the treatment. Moreover, the duration of endocrine treatment with letrozole correlated positively with the chance of obtaining a greater Ki67 decrease. Molecular analyses showed that treatment significantly reduced viable cells. Interestingly, it also appeared to prevent chemotherapy-induced CEP mobilisation, thus interfering with neovascularisation. In addition, patients with higher baseline levels of CEPs were more likely to experience a clinical response.

Further studies investigating optimal timing, duration and combination of antiangiogenic agents with conventional antineoplastic drugs, and particularly the identification of surrogate markers of antiangiogenic activity, are warranted.

## Figures and Tables

**Table 1:** Patient characteristics at baseline

**Characteristics**	**No. of patients**	**%**
Total enrolled/evaluable	37/36	—
		
*Age (years)*		
Median	44	
Range	30–68	
		
*Menopausal status*		
Premenopausal	25	69
Postmenopausal	11	31
		
*Clinical tumour size*		
T2	22	61
T3	9	25
T4	5	14
		
*Clinical nodal status*		
N0	11	31
N1	22	61
N2	1	3
N3	2	5
		
*PgR status*		
PgR negative/low	6	17
PgR positive^a^	30	83
		
*Ki67*		
<20%	14	39
⩾20%	22	61
		
*Nuclear grade*		
1	1	3
2	27	75
3	6	17
Unknown	2	5

PgR=progesterone receptor.

a Positive: ⩾10%.

**Table 2:** Response after treatment

	**No.**	**%**
Evaluable patients	36	
Pathological complete response	0	0
		
*Clinical response*		
Complete	1	3
Partial	30	83
Stable disease	5	14
Progression	0	0
		
*Pathological tumour size*		
T1	15	42
T2	16	44
T3	4	11
T4	1	3
		
*Nodal status at surgery*		
N0	14	39
N1	9	25
N2	8	22
N3	5	14
		
*Type of surgery*		
Breast-conserving surgery	23	64
Mastectomy	13	37

**Table 3:** Toxicities grade ⩾2

	**Grade 2**		**Grade 3**		**Grade 4**	
	***N***	**%**	***N***	**%**	***N***	**%**
Leukopaenia	5	14	5	14	0	(−)
Neutropaenia	4	14	12	14	4	11
Nausea	7	19	0	(−)	0	(−)
Vomiting	3	5	0	(−)	0	(−)
Diarrhoea	2	(−)	0	(−)	0	(−)
Stipsis	8	22	1	3	2	8
Mucositis	7	19	2	5	0	(−)
Biochemical^a^	5	14	3	8	0	(−)
Neurological	0	(−)	1	3	0	(−)
Myalgia	1	3	0	(−)	0	(−)
Asthaenia	5	14	1	3	2	8
Epigastralgia	4	11	0	(−)	0	(−)
HFS	4	11	1	3	1	4
DVT	0	(−)	4	11	0	(−)
Hypertension	7	19	2	5	0	(−)
Proteinuria	1	3	0	(−)	0	(−)
Infection/fever	2/2	5/5	0/2	0/5	0	(−)

DVT=deep venous thrombosis; HFS=hand–foot syndrome.

a Included alteration of liver function (AST, ALT and bilirubin).
